# Ultra-wideband TFLN modulator with selectively removed slab based on multifunctional BCB platform for high coupling efficiency and suppressed EO relaxation

**DOI:** 10.1515/nanoph-2025-0460

**Published:** 2025-12-04

**Authors:** Yutong He, Hao Liu, Changzheng Sun, Bing Xiong, Zhibiao Hao, Jian Wang, Lai Wang, Yanjun Han, Hongtao Li, Lin Gan, Jiyuan Zheng, Yi Luo

**Affiliations:** Beijing National Research Center for Information Science and Technology (BNRist), State Key Laboratory of Space Network and Communications, Department of Electronic Engineering, 12442 Tsinghua University, Beijing 100084, China

**Keywords:** thin-film lithium niobate modulator, BCB-clad edge couplers, low-k underfill, DC bias drift, EO relaxation-free, IM/DD

## Abstract

Thin-film lithium niobate (TFLN) has a proven record of building high-performance electro-optic (EO) modulators. However, it has consistently posed challenges in securing low driving voltage, wide electro-optic bandwidth, low insertion loss, and high modulation efficiency simultaneously. Here, we demonstrate a telecom-wavelength EO modulator on the TFLN platform incorporating multifunctional benzocyclobutene (BCB) material. The low dielectric constant (low-k) BCB effectively reduces RF loss of the modulator and enables perfect velocity matching with a narrow electrode gap, thereby overcoming the conventional voltage–bandwidth trade-off. Meanwhile, in combination with a bilayer inversely tapered waveguide, it also facilitates the realization of high-efficiency edge couplers, significantly reducing the coupling loss of the modulator. In addition, the underlying TFLN slab is selectively removed to eliminate dielectric relaxation, ensuring a stable low-frequency EO response and bias-drift-free operation. The fabricated 13-mm-long modulator exhibits low half-wave voltages V_π_ of 1.5 V in the C-band and 1.19 V in the O-band, corresponding to half-wave voltage-length products of 1.95 V·cm and 1.55 V·cm, respectively. Thanks to the BCB-clad edge coupler, an ultra-low coupling loss of 0.54 dB per facet is obtained. Ultra-wide EO bandwidths exceeding 110 GHz across the C + O-bands are demonstrated, and high-speed PAM8 data transmission with data rates up to 390 Gbit/s is successfully recorded in both C- and O-bands. The proposed modulator architecture not only delivers excellent overall performance, but also simplifies the fabrication process and expands the application potential.

## 1 Introduction

Thin-film lithium niobate (TFLN) photonic integrated circuits have enabled significant advancements in optical science and technology over the past few years [1]. TFLN Mach–Zehnder modulators (MZMs) are particularly attractive for applications including optical communications [2], optical interconnects [3], microwave photonics [4], optical computing [5], frequency metrology [6], and sensing [7]. Thanks to their superior electro-optic (EO) performance, TFLN MZMs are also considered as a promising candidate for next-generation high-speed data transmission [8], [9].

According to the Nyquist criterion, the maximum achievable symbol rate in high-speed transmission systems is fundamentally limited by the EO bandwidth of modulators. Insufficient bandwidth leads to severe inter-symbol interference (ISI) and elevated bit error rate (BER). Achieving a wide EO bandwidth requires low microwave loss in traveling-wave electrodes, perfect microwave-optical velocity matching, and good impedance matching. Approaches such as substrate removal [8], [10], [11], and EO equalization [12] have been proposed to improve the bandwidth, but often at the cost of compromised mechanical stability, increased fabrication complexity, or excessive device length. To overcome these limitations, we have proposed a novel scheme that employs a thick benzocyclobutene (BCB) underfill to simultaneously reduce the microwave loss and tune the microwave refractive index, thereby extending the EO bandwidth [13].

In addition to bandwidth, the insertion loss of EO modulators also has a considerable impact on the overall system performance. To minimize the insertion loss resulting from significant mode-field mismatch between optical fibers and submicron TFLN waveguides, specially designed edge couplers have been widely adopted for their broadband and high-efficiency characteristics. Various schemes have been explored, ranging from single-layer [14] and bilayer [15] inverse taper structures to silicon oxynitride [16]- or SU8 [17]-clad waveguides, substrate-recessed cantilever structures [18], [19], and bident structures [20], demonstrating coupling loss reduced to 0.54 dB per facet. However, practical implementation of these edge couplers on modulators remains challenging, as some schemes involve complex fabrication processes, whereas others suffer from degraded mechanical robustness due to partially suspended waveguides.

Moreover, EO relaxation remains a critical challenge in TFLN modulators. The screening effect of defect charges in TFLN tends to degrade the modulation fidelity at low frequencies, thus limiting their practical applications [21], [22], [23]. Also related to the EO relaxation is the problem of DC bias drift, which has plagued lithium niobate MZMs since their advent. While thermo-optic phase shifters can be adopted for stable bias control [24], [25], the relatively weak thermo-optic coefficient of TFLN results in high power consumption, making such devices unsuitable for transmitter systems with strict power constraints.

In this work, we present a high-performance TFLN MZM with low process complexity, which is based on a multifunctional hybrid TFLN-BCB platform [26] and selective removal of the TFLN slab. The low-k BCB layer, which serves as an underfill beneath the traveling-wave electrodes, effectively reduces the microwave loss at high frequencies and preserves excellent velocity matching. The BCB also allows the formation of BCB-clad edge couplers for the TFLN modulator, which significantly expands the optical mode field for reduced coupling loss. By employing photosensitive BCB, the fabrication process of the clad waveguide is substantially simplified and remains fully compatible with existing modulator fabrication process [13], requiring only standard UV lithography without additional etching steps. Furthermore, a direct metal-lithium niobate sidewall contact is formed via selective removal of the slab to mitigate EO relaxation in TFLN, facilitating stable low-frequency EO response and bias drift-free operation, without compromising modulation efficiency or insertion loss. The fabricated 13-mm-long device demonstrates a low half-wave voltage V_π_ of 1.5 V in the C-band and 1.19 V in the O-band, and a coupling loss of 0.54 dB per facet for transverse-electric (TE) mode. An ultra-wide EO bandwidth with a roll-off of less than 2 dB up to 110 GHz is recorded, which guarantees the high data rates exceeding 390 Gbit/s with low system power consumption.

## 2 Device design and fabrication

**Figure 1:**
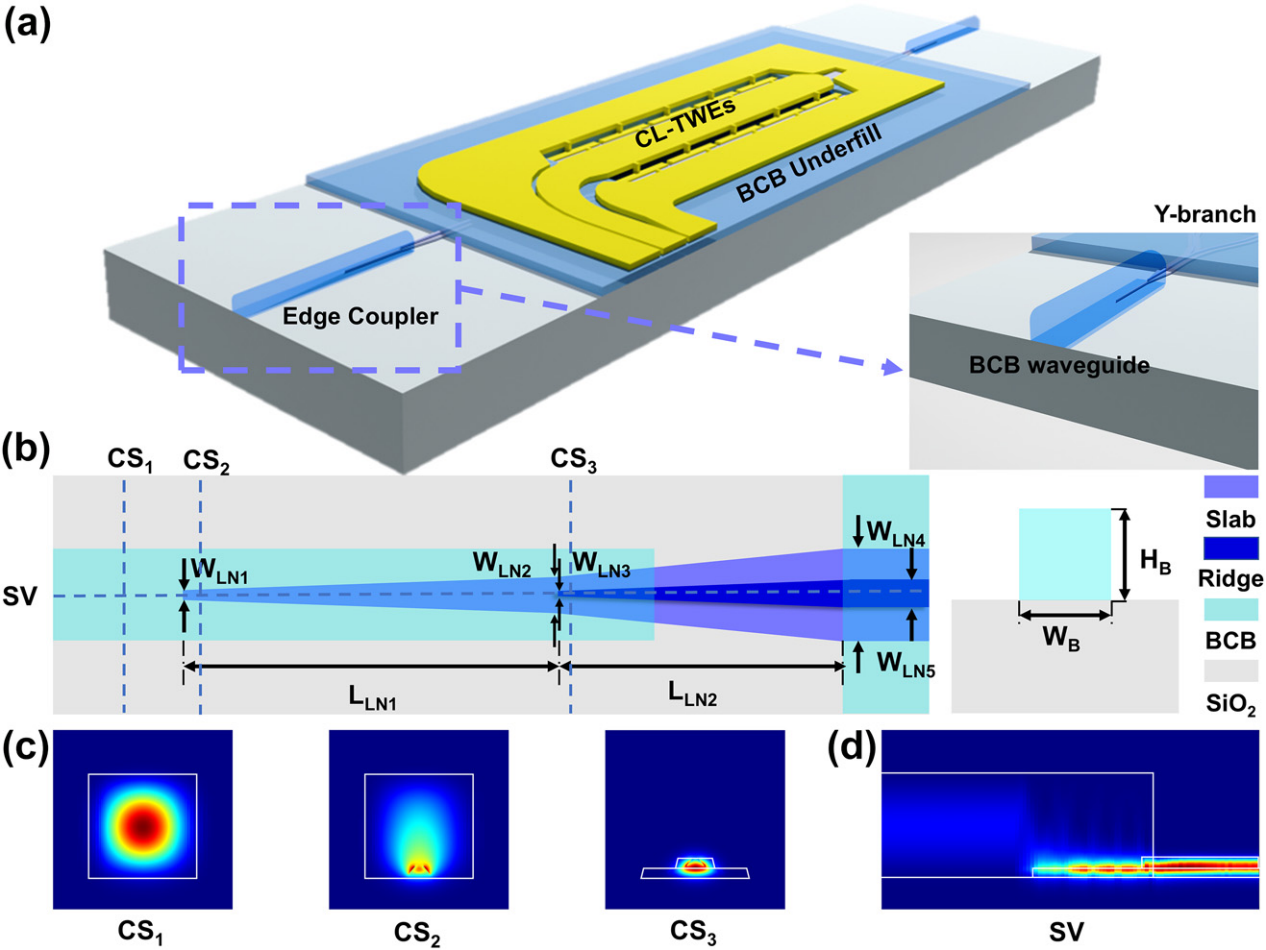
TFLN modulator with BCB-clad edge couplers. (a) 3D schematic of the TFLN modulator integrated with BCB-clad edge couplers. (b) Top view of the BCB-clad edge coupler. (c) Simulated mode profile of the fundamental TE mode at different cross sections (CS). (d) Side-view (SV) of simulated mode propagation within the proposed edge coupler.

The proposed modulator with integrated edge couplers, as illustrated in Figure 1(a), is fabricated on a 400-nm-thick X-cut TFLN layer on top of a 2-μm-thick buried SiO_2_ layer and a 500-μm-thick quartz substrate. Two Y-branches are employed to symmetrically split and recombine the optical signal. The gold electrodes are designed in a periodic capacitively loaded traveling-wave electrode (CL-TWE) configuration [27], [28], combined with BCB underfill for enhanced overall performance of the modulator.

### 2.1 BCB-clad edge couplers

The top-view of the proposed edge coupler is illustrated in Figure 1(b), which consists of a BCB waveguide and a bilayer inversely tapered TFLN waveguide. As BCB exhibits a refractive index of ∼1.56, slightly higher than that of the underlying buried SiO_2_ layer, it forms a weakly confined optical waveguide that expands the mode field diameter for efficient fiber coupling. In addition, the excellent optical transparency of BCB ensures low transmission loss across a broad spectral range, while photosensitive BCB allows direct waveguide fabrication via a single-step lithography process, eliminating the need for dry etching.

**Figure 2:**
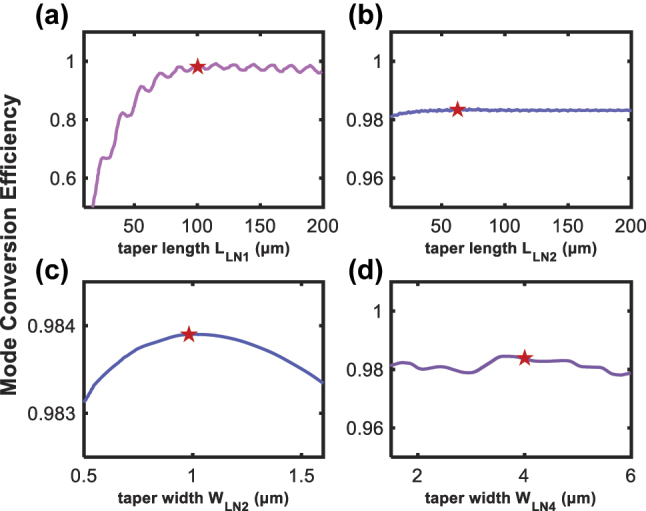
Simulated results of the edge coupler. (a) – (d) Simulated mode conversion efficiency versus length and width of the bilayer inverse taper, with the red stars indicating the designed parameter values of the coupler.

To achieve optimal mode matching with a 3.5-μm mode field diameter (MFD) lensed fiber, the width (W_B_) and thickness (H_B_) of the BCB waveguide are designed to be 3.2 μm and 2.8 μm, respectively, based on finite difference eigenmode (FDE) simulations. The bilayer inverse tapers are engineered to adiabatically couple light from the BCB waveguide to the submicron TFLN waveguide. Taking fabrication tolerances into account, the tip widths of both the top and bottom tapers, denoted as W_LN1_ and W_LN3_, are set to 150 nm, which are sufficiently narrow to expand the effective mode area at the coupling interface for improved coupling efficiency. In addition, the upper TFLN ridge waveguide is defined by a 200-nm etch into a 400-nm-thick TFLN film, providing robust single-mode optical confinement. Figure 2 illustrates the influence of taper dimensions on mode conversion efficiency in the bilayer structure. Simulation results reveal that the efficiency is primarily determined by the length of the bottom taper. Balancing device compactness and coupling efficiency, the bottom and top taper lengths (L_LN1_ and L_LN2_) are set to 100 μm and 60 μm, respectively, with a bottom taper width W_LN2_ of 1 μm. The output bottom taper width W_LN4_ is designed to be 4 μm to ensure both alignment tolerance and fabrication robustness. The simulated total coupling loss is approximately 0.4 dB per facet, which is primarily attributed to Fresnel reflection at the fiber-waveguide interface, whereas the mode conversion loss is as low as 0.06 dB.

Figure 1(c) presents the optical field distributions of the fundamental TE mode at different cross-sections of the designed edge coupler, while Figure 1(d) illustrates the side view of optical transmission along the entire coupling structure. The input light is initially guided via a low-loss, single-mode BCB waveguide and then adiabatically transferred into the bottom layer of the bilayer inverse taper. The optical mode size is significantly expanded by the weakly confined BCB-clad waveguide, facilitating efficient coupling with the lensed fiber. As the light propagates, it gradually transitions from the bottom taper to the top taper over the designed length of L_LN2_. The light is subsequently fed into the modulation region through a single-mode TFLN waveguide, where EO interaction takes place.

### 2.2 Modulation section

**Figure 3:**
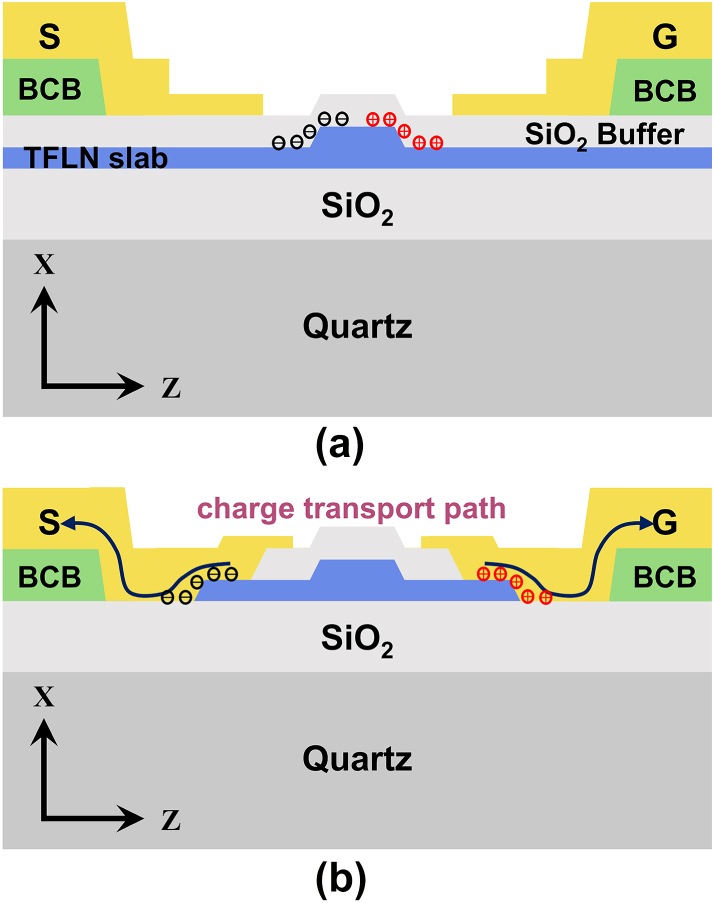
Schematic of two TFLN modulator structures with conventional and selectively slab-removed waveguides. (a) Conventional lithium niobate waveguide and electrode structure with a silicon oxide isolation layer. (b) TFLN waveguide with selectively removed slab for eliminating low-frequency relaxation.

EO relaxation in TFLN poses a critical challenge to the practical deployment of LN-based modulators [21], [22], [23]. This phenomenon arises from the screening effect of free charge carriers within and around the TFLN material. These defect charges accumulate along the waveguide sidewalls, forming an interfacial conductive layer that shields the externally applied low-frequency electric field. To mitigate this effect, instead of the conventional waveguide, a waveguide with selectively removed slab is adopted in our design, as shown in Figure 3. This design removes most of the bottom TFLN slab in non-critical regions. Additionally, the SiO_2_ cladding is also partially removed, enabling direct electrical contact between the electrodes and the Z-plane sidewalls of the TFLN, providing a conductive path for the accumulated defect charges, thereby mitigating the low-frequency electric field screening effect.

Based on this selectively removed slab architecture, a systematic optimization has been conducted on waveguide and electrode parameters. According to finite-element-method (FEM) based simulations, for retained slab width exceeding 4 μm, the mode confinement and the metal-induced optical absorption are basically the same as those of a conventional waveguide. A narrow electrode gap of 3.5 μm and a 300-nm-thick SiO_2_ cladding are adopted to achieve high modulation efficiency and low metal-induced optical loss simultaneously [27]. As illustrated in Figure 1(a), the modulation electrodes adopt CL-TWE architecture. A thick layer of BCB, featuring a low dielectric constant of 2.65, is employed as a low-k underfill beneath the main electrode region of the CL-TWEs, with a thickness identical to that of the BCB-clad waveguide in the edge couplers. The thick BCB layer also lies beneath the bent electrodes over the TFLN waveguides, thus suppressing the metal-induced absorption loss. Precise velocity matching, which is critical for extending EO bandwidth, is secured by judicious optimization of the structural parameters of the CL-TWEs [13].

### 2.3 Device fabrication

**Figure 4:**
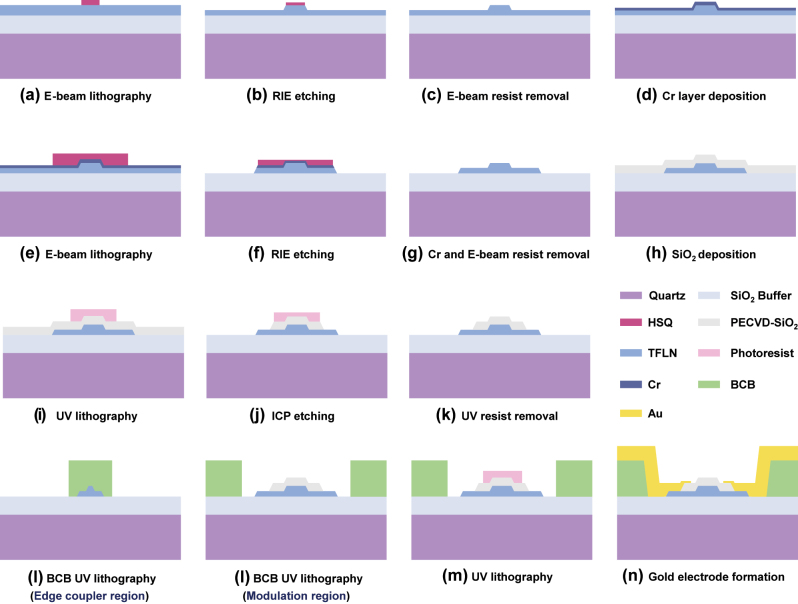
The complete device fabrication process.

**Figure 5:**
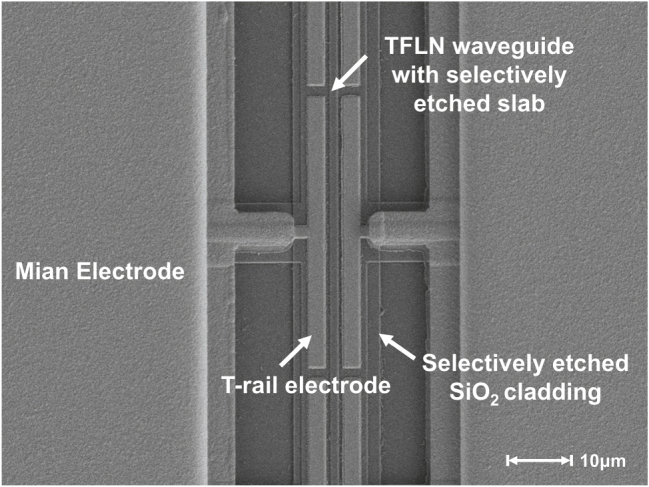
SEM image of the fabricated CL-TWEs and TFLN waveguide with selectively removed slab.

The fabrication process of the proposed TFLN modulator is detailed in Figure 4. Both the bilayer inverse taper and the TFLN waveguides are patterned using electron beam lithography (EBL), followed by argon-based reactive ion etching (RIE). Following the formation of the upper waveguide with hydrogen silsesquioxane (HSQ) resist as the hard mask, a chromium (Cr) adhesion layer is deposited via magnetic sputtering prior to a second HSQ layer coating. During the subsequent etching step, the pattern is first transferred to the Cr layer and then etched into the TFLN layer to form the bottom waveguide. Hydrochloric acid (HCl) is used to remove the etch mask instead of HF, which avoids damage to the underlying SiO_2_, quartz substrate, and lithium niobate thin film. Notably, the Cr layer also eliminates the charging effects on TFLN surface during the EBL exposure. Next, a 300-nm-thick SiO_2_ buffer layer is deposited over the TFLN waveguide, which helps reduce the metal-induced optical absorption loss due to the narrow gap electrodes. The buffer layer away from the ridge region is then removed by inductively coupled plasma (ICP) etching, so as to facilitate direct electrical contact between the metal electrodes and the TFLN waveguide. Subsequently, a 2.8-μm-thick photosensitive BCB is spin-coated and patterned through a one-step UV lithography. This multifunctional BCB simultaneously serves as the cladding of the edge coupler and as the low-k underfill beneath the CL-TWEs. Finally, the 1.7-μm-thick gold CL-TWEs are fabricated using a lift-off process combined with electroplating. Scanning electron microscope (SEM) image shown in Figure 5 reveals the fabricated CL-TWEs and TFLN waveguide with selectively removed slab.

## 3 Device performance characterization

### 3.1 Optical coupling performance of edge coupler

**Figure 6:**
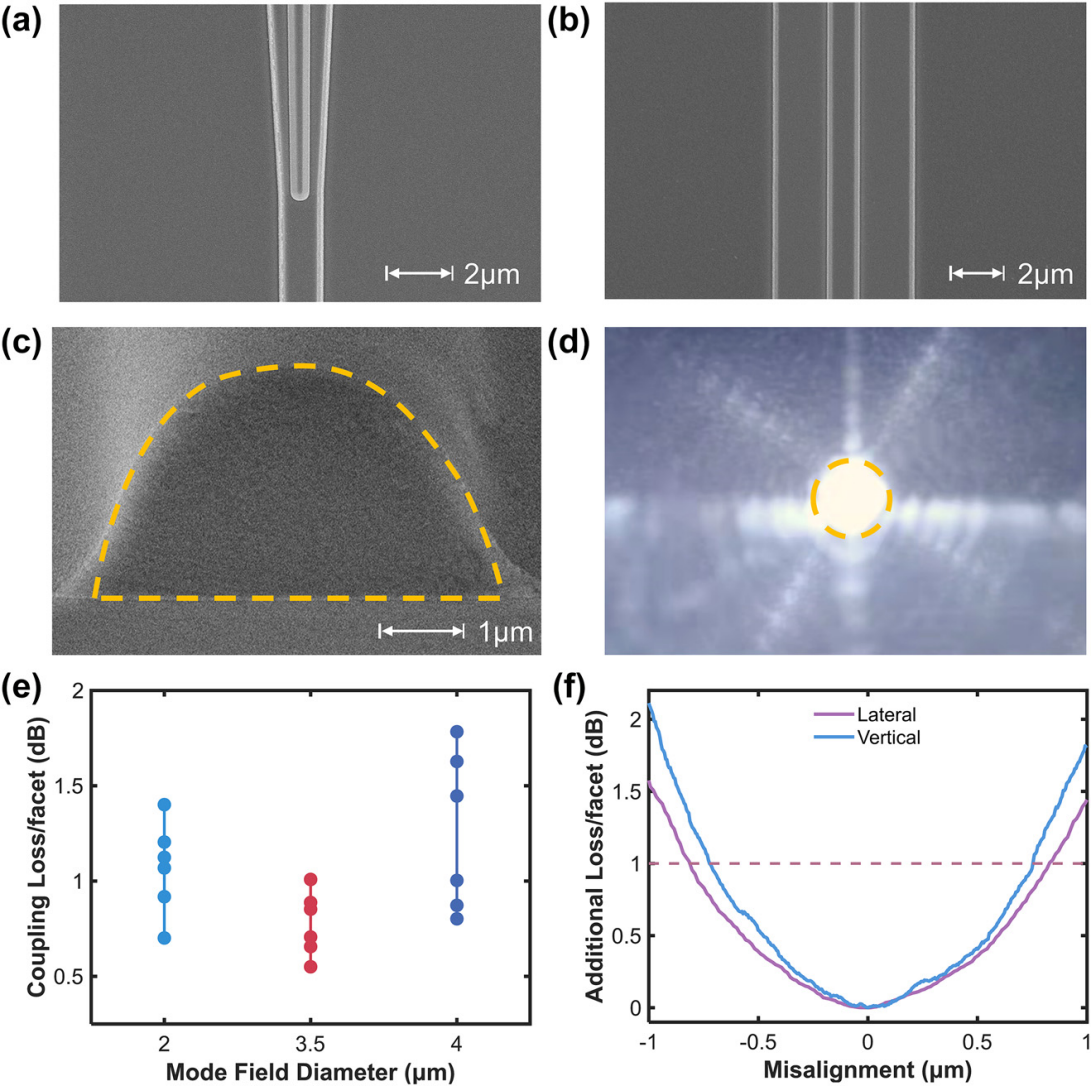
SEM images of (a) the taper tip, (b) the TFLN waveguide with selectively removed slab, and (c) the end facet of the BCB waveguide. (d) The output spot of the BCB-clad edge coupler. (e) Coupling losses of the fabricated edge couplers with fibers of different MFDs at 1,550 nm. (f) Alignment tolerance of the coupler with a 3.5-μm-MFD fiber.

Figure 6(a) and (b) show the SEM images of the bilayer taper and the TFLN waveguide with selectively removed slab, while Figure 6(c) reveals the polished end facet of the BCB waveguide. The output spot from the edge coupler for the fundamental TE mode captured with an infrared CCD camera is shown in Figure 6(d).

The coupling loss of the edge coupler at 1,550 nm was derived from the propagation loss measurement of straight TFLN waveguides with integrated edge couplers. As illustrated in Figure 6(e), with a 3.5-μm-MFD lensed fiber, the minimum coupling loss for TE polarization is 0.54 dB per facet, while the mean value across different couplers is 0.77 dB per facet. Variations in the coupling loss are attributed primarily to misalignments between the BCB waveguide and the bilayer taper, likely stemming from overlay inaccuracies between the EBL and the UV lithography processes. Coupling tests for lensed fibers with different MFDs further demonstrate that the optimal coupling occurs with the 3.5-μm-MFD fiber, thereby verifying the consistency between the design and the fabrication results.

In addition, alignment tolerance between the edge coupler and the 3.5-μm-MFD lensed fiber was characterized by shifting the fiber laterally and vertically using a precision motorized stage. As shown in Figure 6(f), a 1-dB alignment tolerance was recorded for a lateral displacement of ±0.8 μm or a vertical displacement of ±0.75 μm.

The total insertion loss of our modulator equipped with two BCB-clad edge couplers is measured to be 6.3 dB in the C-band. The waveguide propagation loss, characterized with the Fabry–Perot (FP) method [29], is approximately 2.2 dB/cm, amounting to 3.96 dB loss for a total TFLN waveguide length of 18 mm. The metal-induced absorption loss is estimated to be roughly 0.68 dB/cm, or 0.88 dB loss for the 13-mm-long modulation region. As a result, the coupling loss is inferred to be 0.73 dB/facet, which agrees well with the measured mean value of 0.77 dB/facet.

In the O-band, the modulator exhibits a total insertion loss of 9.3 dB. The measured waveguide propagation loss is 2.84 dB/cm, the metal absorption loss is 0.22 dB/cm, while the coupling loss is 1.95 dB/facet, which is consistent with the simulated value of 1.74 dB/facet at 1,310 nm. The slightly higher coupling loss compared with that in the C-band primarily arises from the larger mode mismatch at 1,310 nm, since our BCB-clad edge coupler is mainly optimized for operation around 1,550 nm.

### 3.2 Low-frequency electro-optic response

**Figure 7:**
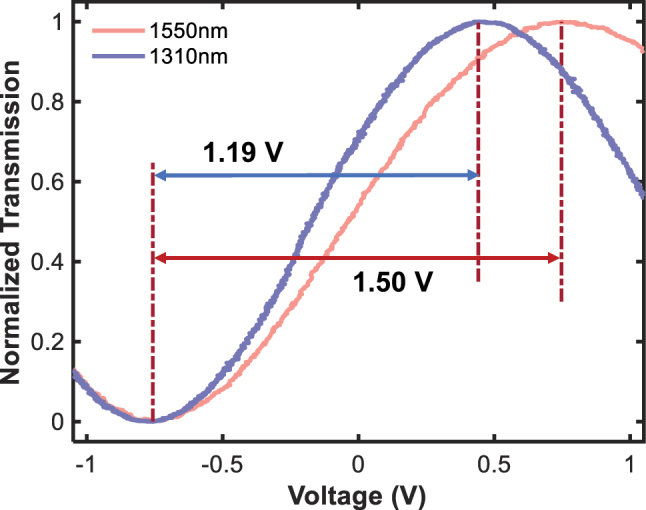
Normalized optical transmission as a function of driving voltage for a 13-mm-long TFLN modulator.

To determine the half-wave voltage of the modulator, a continuous-wave laser at 1,550 nm or 1,310 nm was used as the input light, and a polarization controller was employed to ensure TE-polarized input. A 1 MHz triangular waveform was applied to the modulator with a modulation length of 13 mm, while the output optical signals were captured by a photodetector and monitored using a real-time oscilloscope (Tektronix MDO4034C). The normalized transmission curves illustrated in Figure 7 reveal half-wave voltages V_π_ of 1.5 V in the C-band and 1.19 V in the O-band, yielding a low V_π_·L of 1.95 V·cm and 1.55 V·cm, respectively. The improved modulation efficiency in the O-band arises from enhanced phase accumulation at shorter wavelengths.

**Figure 8:**
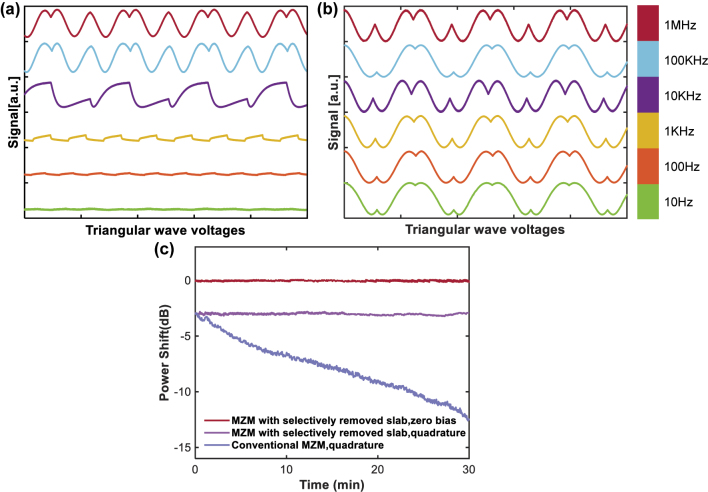
Low-frequency electro-optic modulation curves of (a) a conventional TFLN modulator and (b) a TFLN modulator with selectively removed slab under triangular waveforms at various frequencies. Curves in different colors correspond to different modulation frequencies. (c) Output power variations over time. All tests are carried out at room temperature.

To further evaluate the low-frequency electro-optic responses, a series of triangular voltage waveforms with frequencies varying from 1 MHz to 10 Hz were applied to two different MZMs with equal length fabricated on the same wafer. As shown in Figure 8(a), for conventional waveguide structure, significant modulation waveform distortion is observed at frequencies below 100 kHz. In contrast, modulator incorporating selectively removed slab exhibits undistorted and stable modulation responses within the entire tested frequency range, as illustrated in Figure 8(b). This observation underscores the effectiveness of slab removal in mitigating the screening effect and preserving low-frequency modulation fidelity.

In addition, Figure 8(c) compares the output optical power variations over time. The modulator with selectively removed slab exhibits extremely low power fluctuations, whereas the conventional modulator suffers from a significant drift. Compared with thermal-optic phase shifters [24], [25], which circumvent the DC bias drift in TFLN MZMs at the expense of increased power consumption, our approach provides a simpler and more energy-efficient solution for achieving a stable DC-bias pre-set.

### 3.3 High-frequency electro-optic response

**Figure 9:**
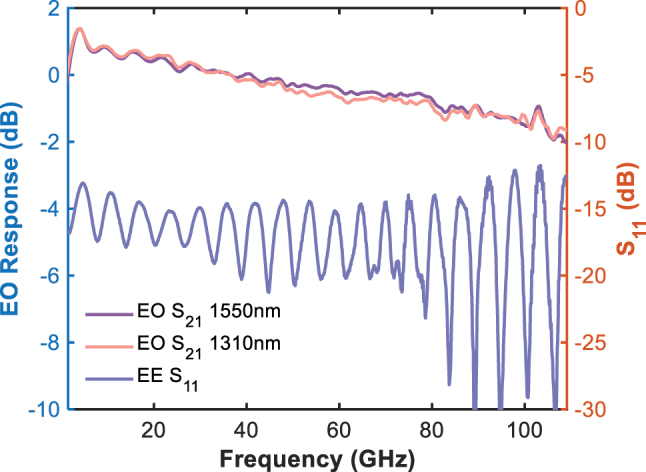
Measured electro-optic responses at 1,550 nm and 1,310 nm and electrical reflection S_11_ up to 110 GHz.

The high-frequency EO responses of the modulator with on-chip terminal resistor in the C- and O-bands were characterized using a 110 GHz lightwave component analyzer (LCA, Keysight N4372E). To ensure the accuracy and reliability of the measurements, standard de-embedding procedures were rigorously performed to remove the effects of RF probes, coaxial cables, and photodetectors from the test results. As shown in Figure 9, the device exhibits ultra-flat EO responses, with roll-offs of only ∼2 dB up to 110 GHz, demonstrating excellent high-speed modulation performance. The electrical reflection S_11_ remains below −13 dB across the entire frequency range, indicating good impedance matching. Thanks to the low microwave loss enabled by the low-k BCB underfill beneath the CL-TWEs, and consistent RF-optical velocity matching (optical group velocity n_g_ = 2.24@1,550 nm and 2.26@1,310 nm), our modulator exhibits outstanding bandwidth performance in both C- and O-bands.

### 3.4 High-speed data transmission

In order to verify the high-speed data transmission capability of our high-performance TFLN modulator, a series of eye-diagram measurements were carried out in an intensity modulation/direct detection (IM/DD) system [30]. On the transmitter side, pulse-amplitude modulation (PAM) signals were generated by a 256 GSa/s arbitrary waveform generator (AWG, Keysight M8199A), subsequently amplified by a 67 GHz electrical amplifier, passed through a 65 GHz bias tee, and ultimately delivered to the modulator via a 67 GHz ground-signal-ground (GSG) probe. On the receiver side, the modulated optical signals were captured using a digital sampling oscilloscope (DSO) equipped with a 65 GHz optical sampling module (Keysight N1030A). To compensate for the RF attenuation throughout the entire electrical link, a pre-emphasis filter was applied to the driving signals at the transmitter.

**Figure 10:**
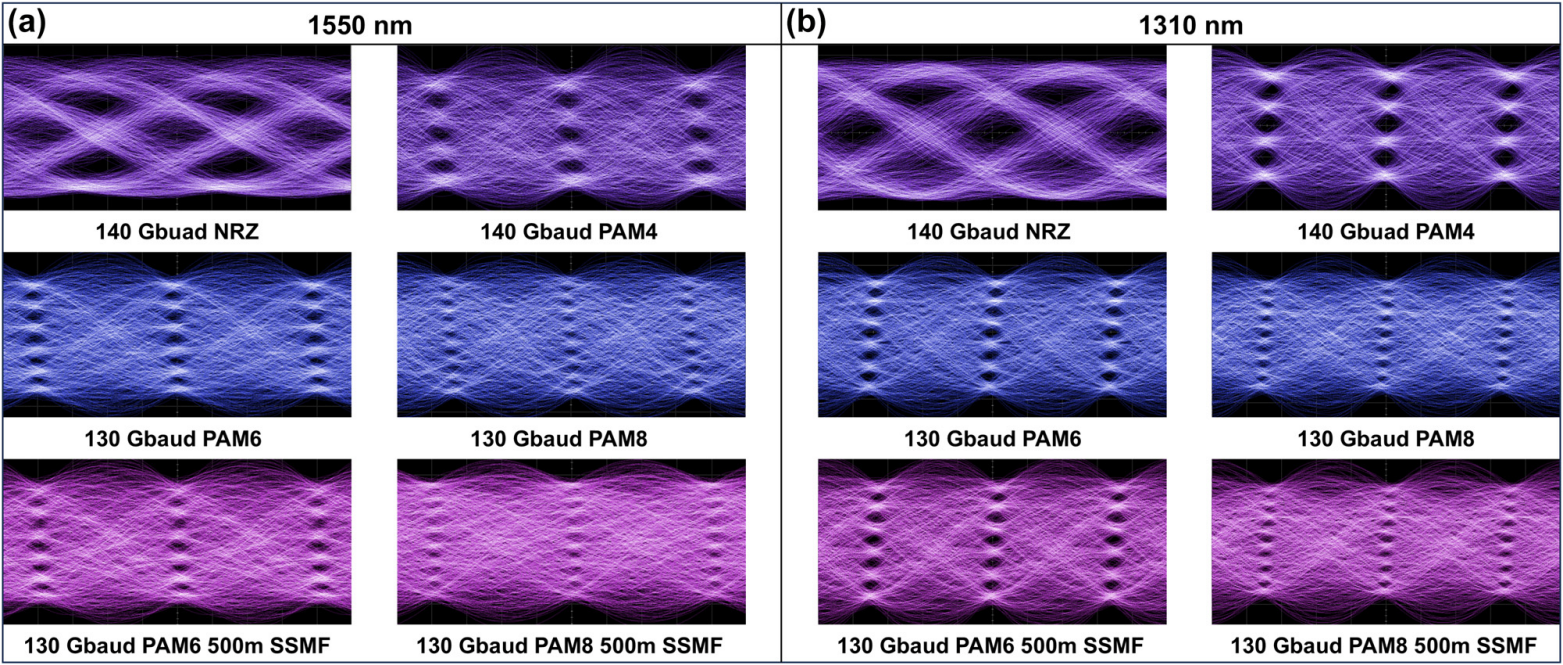
Measured eye diagrams in (a) C-band and (b) O-band for back-to-back transmission of 140 Gbaud NRZ and PAM4, 130 Gbaud PAM6 and PAM8 signals, as well as 500 m SSMF transmission of 130 Gbaud PAM6 and PAM8 signals.

Thanks to the ultrahigh bandwidth and broad operation spectrum, clear eye diagrams were recorded across both C- and O-bands, successfully demonstrating data transmission of 140 Gbaud NRZ, 140 Gbaud PAM4, 130 Gbaud PAM6, and 130 Gbaud PAM8, corresponding to a maximum data rate of 390 Gbit/s. The maximum baud rate demonstrated in these measurements is currently limited by the bandwidth of the electrical components employed in the transmission system, including the AWG, electrical amplifier, bias tee, and DSO. Specifically, as shown in Figure 10, opened eyes were observed for both back-to-back (B2B) and 500-m standard single-mode fiber (SSMF) transmission scenarios. The eye diagram in the C-band deteriorates considerably after 500 m of fiber transmission due to dispersion at 1,550 nm, whereas it remains almost unvaried in the O-band owing to the near-zero dispersion of SSMF around 1,310 nm. This phenomenon could be mitigated in future work through integration with on-chip dispersion compensation devices [31].

In addition to high data rates, the 13-mm-long modulator exhibits highly competitive low-power performance, benefiting from its low half-wave voltage. The energy consumption per bit can be expressed as *E*_bit_ = *V*_RMS_^2^/(*B·R*), where *V*_RMS_ is the root-mean-square drive voltage, *B* is the bit-rate, and *R* is the terminal impedance [32]. It requires only 245 mV (C-band) and 146 mV (O-band) RMS driving voltages, corresponding to electrical energy consumptions of 4.87 fJ/bit and 1.74 fJ/bit, respectively, which is well below the typically 10–100 fJ/bit range of traditional TFLN modulators [33], [34].

Table 1 summarizes the performance metrics of recently reported TFLN modulators. Our modulator delivers superior overall performance, featuring a 1 V-level half-wave voltage, ultrawide EO bandwidth, and ultrahigh-speed data transmission capability for IM-DD systems, while maintaining ultralow energy consumption.

**Table 1:** Performance comparison of TFLN modulators.

References	Optical band	*V*_π_ [V]	*V*_π_·*L* [V·cm]	3-dB BW [GHz]	Data rates [Gbit/s]	Coupling loss [dB/facet]	Energy consumption [fJ/bit]	Characteristic
2018 [32]	1,550	1.4	2.8	>45	210(PAM8)	5	14	CPW/Si_sub
2018 [35]	1,550	3.8	3.8	−19@500	–	–	–	Phase modulator
2020 [36]	1,550	1.3	3.12	29	–	–	–	Si_3_N_4_ on TFLN
2021 [28]	1,550	1.3	2.6	−1.8@50G	–	6	–	CL-TWEs/quartz_sub
2021 [27]	1,550	3.4	1.7	−2@67G	–	–	–	CL-TWEs/quartz_sub
2021 [17]	1,550	2.36	3.068	60	200(DMT)	0.5	–	SU8-clad
2022 [8]	1,550	2.2	2.2	−1.4@67G	112(PAM4)	–	–	Si_sub/undercuting
2023 [33]	1,550	4.4	2.2	84	240(PAM8)	–	124	Waveguide placed nonsymmetrically
2023 [3]	1,550	4.74	2.37	110	250(PAM6)	6.5	–	CPW/Si_sub
2023 [37]	1,550	3.52	1.41	>67	64(OOK)	3.3	–	CPW/high-k
2024 [23]	456	0.63	0.38	6.9	–	–	–	Blue light
2024 [34]	1,547.1	2.1	0.21	>110	240(PAM4) 300(PAM8)	–	44 (240 Gbps)	Topologic/slow-light
2024 [38]	1,310	2.04	1.02	108	224(PAM4)	7	–	CPW/ITO
2024 [11]	1,550	2.18	2.18	>67	112(PAM4)	4	–	Si_sub/backholes
2025 [39]	1,550	1	2.0	125	–	>10	–	CL-TWEs/quartz
2025 [40]	1,550	0.52	3.9	10	–	>10	–	Folded/phase modulator
2025 [13]	1,550	1.9	1.33	−0.77@110G	390(PAM8)	6.4	4.42	CL-TWEs/BCB-TFLN
	1,310	1.54	1.08	−0.83@110G	390(PAM8)	–	0.69	
This work	1,550	1.5	1.95	−2@110G	390(PAM8)	0.54	4.87	Relaxation-free/BCB-clad
	1,310	1.19	1.55	−2@110G	390(PAM8)	1	1.74	

## 4 Conclusions

We have developed a high-performance EO modulator with selectively removed slab based on hybrid TFLN-BCB platform, providing a robust and versatile foundation for next-generation TFLN modulators. By leveraging the low dielectric constant and optical transparency of photosensitive BCB, the TFLN modulator achieves superior performance metrics, including a coupling loss as low as 0.54 dB per facet, a low half-wave voltage *V*_π_ of 1.5 V in the C-band and 1.19 V in the O-band, and an ultrahigh bandwidth exceeding 110 GHz. A transmission capacity of 130 Gbaud PAM8 is recorded in an IM/DD system, demonstrating a maximum data rate of 390 Gbps, which is primarily limited by the electrical bandwidth of the measurement setup. The incorporation of waveguide with selectively removed slab further enhances the device characteristics by effectively resolving the long-standing issue of low-frequency EO relaxation and DC bias drift in TFLN modulators. This architecture enables stable low-frequency modulation down to 10 Hz without compromising other performance aspects, while maintaining full compatibility with existing fabrication processes. Overall, the integration of novel multifunctional BCB platform with selectively removed slab scheme establishes a new performance benchmark for TFLN modulators.
